# Circulating levels of inflammatory markers and DNA methylation, an analysis of repeated samples from a population based cohort

**DOI:** 10.1080/15592294.2019.1603962

**Published:** 2019-04-29

**Authors:** Robin Myte, Anneli Sundkvist, Bethany Van Guelpen, Sophia Harlid

**Affiliations:** a Department of Radiation Sciences, Oncology, Umeå University, Umeå, Sweden; b Wallenberg Centre for Molecular Medicine, Umeå University, Umeå, Sweden

**Keywords:** DNA methylation, inflammation, biomarkers, C - reactive protein, colorectal cancer, risk factors, epigenetics, proteomics

## Abstract

DNA methylation in blood may adapt to conditions affecting our health, such as inflammation, and multiple studies have identified differential DNA methylation related to smoking, obesity and various diseases. The purpose of this study was to evaluate previously reported, and explore possible new, associations between levels of inflammatory markers and DNA methylation in blood. We used a well-characterized study population consisting of 127 individuals, all of whom were participants in the population-based Västerbotten Intervention Programme cohort and had provided two blood samples, ten years apart. Levels of CRP and 160 other proteins were measured in plasma, and DNA methylation levels (assessed using the 850K Illumina Infinium MethylationEPIC BeadChip) were measured in white blood cell DNA. Associations between CpG methylation and protein levels were estimated using linear mixed models. In the study we were able to confirm the direction for 85 of 102 previously reported protein-methylation associations. Depicting associations in a network allowed us to identify CpG sites with associations to multiple proteins, and ten CpG sites were each associated with three or more inflammatory markers. Furthermore, two genetic regions included nine additional unreported CpG sites that may represent trans-acting methylation sites. Our study supports a complex interaction between DNA methylation and circulating proteins involved in the inflammatory response. The notion of trans-acting methylation sites affecting, or being affected by, the expression of genes on completely different chromosomes should be taken into account when interpreting results from epigenome-wide association studies.

## Introduction

The connection between chronic low grade inflammation and non-communicable diseases such as diabetes, cardiovascular disease and cancer is well established [1]. Inflammation is an important contributor to carcinogenesis and DNA methylation, which can affect gene expression [2]. Methylation, in turn, has been implicated as a potential mediator between the inflammatory response and subsequent disease. Supporting this, differential DNA methylation is observed in a number of inflammation-related diseases [3–5].

The use of large panels of inflammatory markers may be central to achieving a better understanding of the relationship between inflammation and disease mechanisms, including DNA methylation. Although more than 900 biomarkers of inflammation have been described in the literature [6], there is no clear consensus as to how to measure and quantify chronic low-grade inflammation or which markers can best discriminate between different inflammatory responses [7]. For example, C-reactive protein (CRP), measured by high sensitivity assays, is often used as an unspecific marker of inflammation, but CRP levels are strongly influenced by genetic variants [8] and can be subject to environmental influence, such as smoking or other underlying conditions.

It is also important, but difficult, to elucidate the temporal relationship between inflammation and methylation, as differences in DNA methylation levels could be caused by or be a causal factor driving the inflammatory state. However, analyses of repeated blood samples in observational studies are rare. To our knowledge, no study has investigation methylation and inflammation in a longitudinal setting.

A recent meta-analysis [9] identified 218 potential DNA methylation markers associated with circulating levels of the inflammatory marker CRP, of which 58 were replicated in an independent population of a different ethnic origin. Also, a panel of protein biomarkers (including a large number affecting inflammation) were recently evaluated in relation to DNA methylation and genetic data in another recent study of over 600 samples [10].

The aim of this study was to replicate and expand upon previously reported associations between CpG sites and inflammation, using a set of 127 participants from a population-based cohort, each with two blood samples collected ten years apart. All samples were analyzed for DNA methylation using the Illumina Infinium MethylationEPIC BeadChip array and for plasma proteins using two large pre-designed immunoassay panels containing over 160 plasma protein biomarkers, as well as had extensive phenotype data available for multivariable analysis. CpG sites that replicated within and between the studies, representing highly robust associations with protein biomarkers, were then linked in a network, illuminating some of the underlying connections between inflammation, protein markers and DNA methylation.

## Results

### Baseline and repeat characteristics

Sixty-nine men and 51 women with two measurements, taken approximately 10 years apart, were included in the study (Table 1). The study population was originally selected as part of a project investigating biomarkers for risk prediction and early detection of colorectal cancer, as previously described [11]. Thirty-one participants (26%) were current smokers at baseline. This decreased to 17 (14%) at the repeat sampling, but with weak evidence of a real change (P = 0.08). Both BMI and levels of inflammatory markers generally increased over time (Table 1).

**Table 1.** Characteristics of the study participants at the baseline and repeat measurements.

Variable^a^	Baseline (n = 127)	Repeat (n = 127)	P^b^
Cases/controls	63/64	63/64	-
Sex, male (%)	74 (58)	74 (58)	-
Age (years)	50.0 (40.3–50.2)	59.9 (50.2–60.1)	-
Current smoker	35 (28)	21 (17)	0.10
BMI (kg/m^2^)	25.3 (23.0–27.2)	26.0 (23.8–28.9)	1.5e-08
CRP (mg/L)	0.8 (0.4–1.8)	1.3 (0.7–2.9)	6.2e-05
Proteins (NPX values)^c^			
CCL19	8.8 (8.4–9.2)	8.9 (8.4–9.4)	0.24
CCL4	5.5 (5.2–5.8)	5.6 (5.3–5.9)	0.00027
CX3CL1	6.0 (5.8–6.3)	6.1 (5.9–6.4)	0.00011
CXCL1	8.4 (8.0–8.8)	8.4 (7.8–8.8)	0.18
CXCL11	6.4 (6.0–7.1)	6.7 (6.2–7.3)	0.00038
CXCL13	7.6 (7.2–7.9)	7.7 (7.2–8.0)	0.01
CXCL9	7.0 (6.6–7.5)	7.5 (7.1–8.0)	1.6e-12
Flt3L	8.2 (8.0–8.4)	8.4 (8.1–8.6)	1.2e-09
MIA	9.5 (9.4–9.6)	9.5 (9.3–9.7)	0.068
TNFRSF4	2.7 (2.5–2.9)	2.8 (2.6–3.1)	0.00031
WFDC2	6.5 (6.3–6.7)	6.7 (6.5–6.9)	2.4e-10

BMI: Body mass index, CRP: C-reactive protein, NPX: Normalized protein expression. a Median (interquartile range) for continuous variables, number of participants (%) for categorical variables b Paired Wilcoxon signed rank test for changes in continuous variables, chi-square tests for categorical variables. c Proteins with a significant association to DNA methylation sites in Ahsan et al. [10].

### Replication of previously reported associations

**Figure 1.**
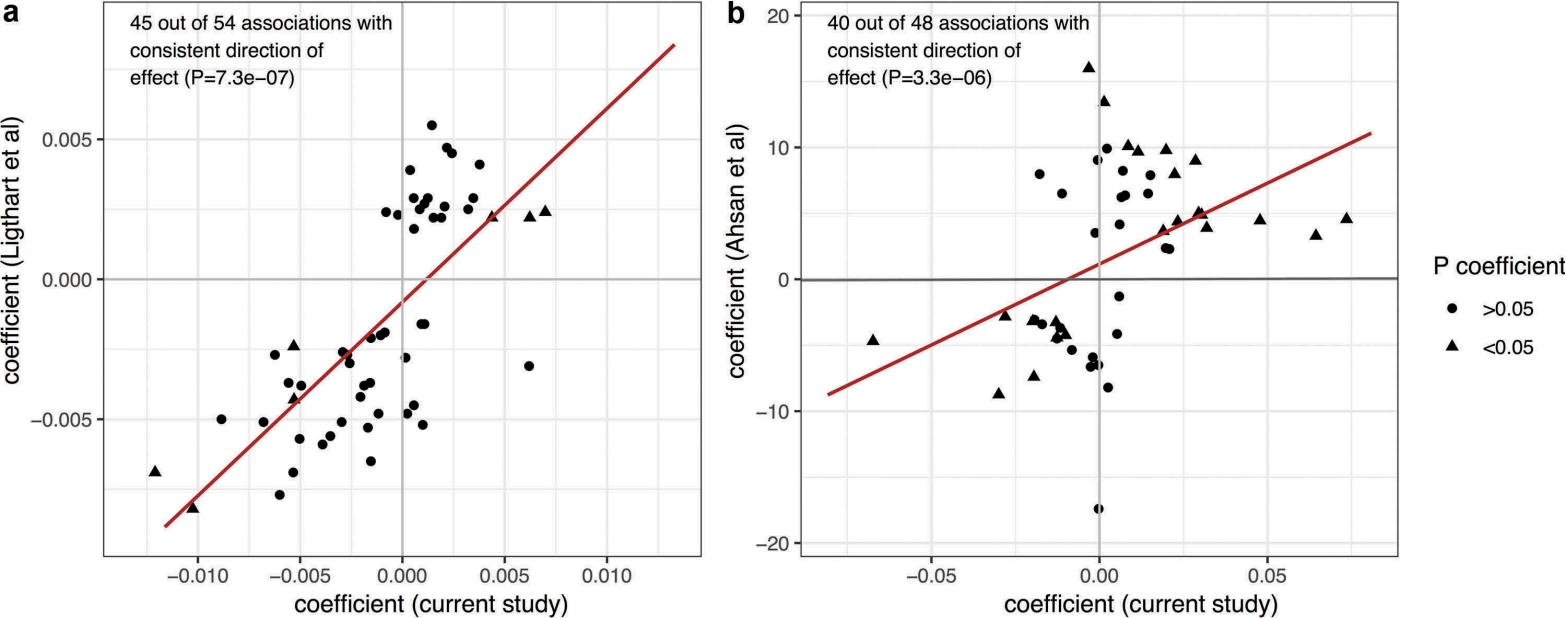
Replication of associations between DNA methylation in blood and CRP and other circulating inflammatory markers. Each point represents the beta coefficient for a previously reported association between an inflammatory marker and methylation at a CpG site (y-axis) in relation to the beta coefficient for the same association in the current study (x-axis). In (a) Ligthart et al. [9], methylation values were modelled as the outcome, and in (b) Ahsan et al. [10], methylation values were modelled as the exposure. The red line represents an estimated regression line. NPX: Normalized protein expression.

We attempted replication of 102 previously reported associations between DNA methylation at 95 CpG sites and levels of 12 plasma proteins (including CRP) [9,10] (Supplementary Table 1). In the present study, 85 associations retained consistent direction of association between protein and methylation levels (P = 7.3*10^−7^, Figure 1(a), P = 3.3*10^−6^, Figure 1(b)), 79 of which represented unique CpG sites (six CpG sites were associated with circulating levels of two different proteins). Two adjacent CpG sites (cg12054453 and cg16936953) in the *WMP1* gene were associated with both CRP levels and CXCL13 levels. Furthermore, four CpG sites (cg05304729, cg09801824, cg07839457 and cg16411857) were associated with circulating levels of both CXCL9 and CXCL11, and two of these sites (cg07839457 and cg164118579) were situated in the same gene (*NLRC5*).

### Within-individual changes in DNA methylation over time

**Figure 2.**
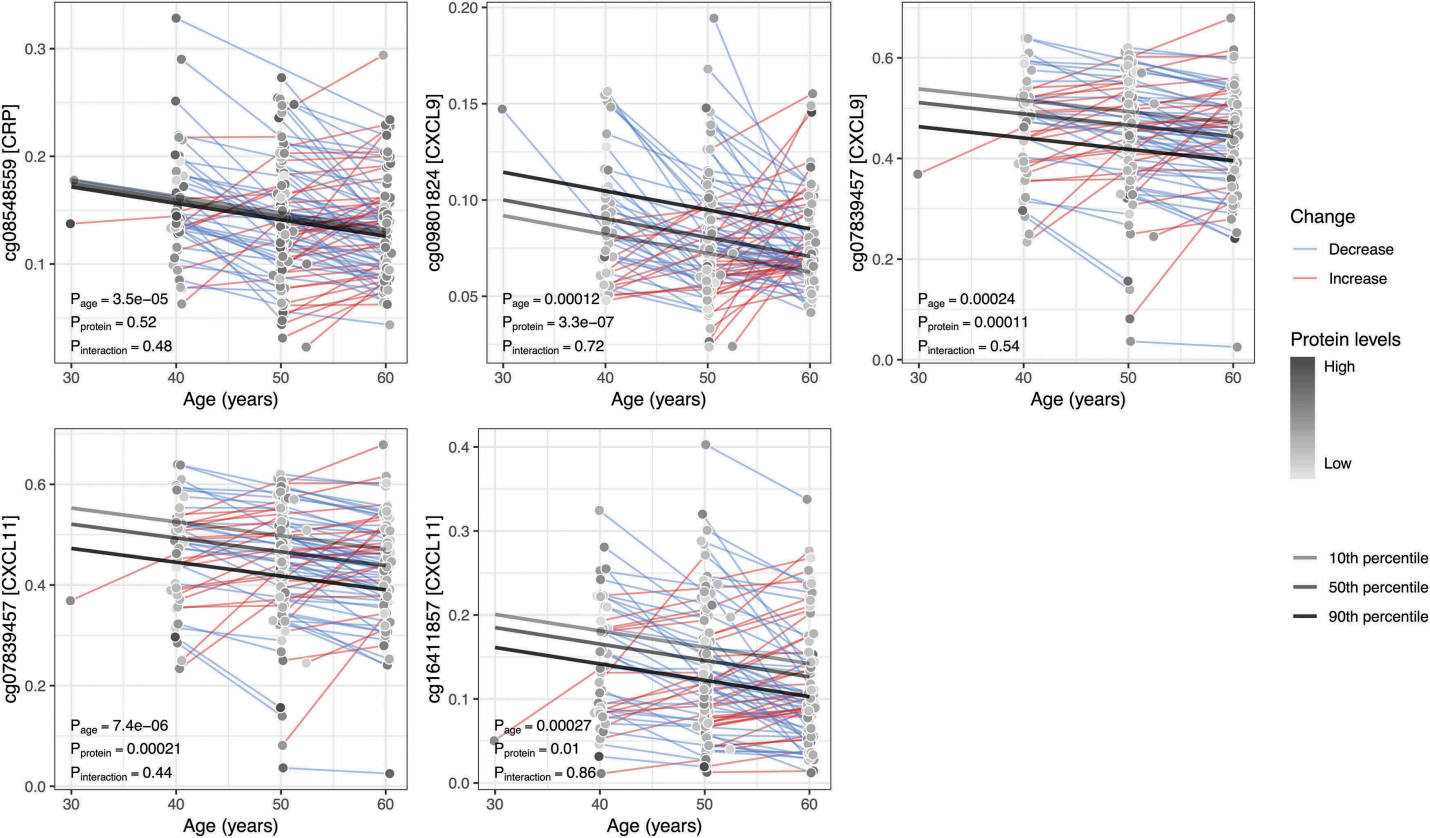
Average within-individual age trajectories for CpG methylation levels. The CpG sites shown demonstrated an association between methylation and levels of an inflammatory protein (in square brackets on the y-axis), as well as a significant change over time (P < 0.0005). Average within-individual age trajectories were estimated in mixed models, including interaction terms between age and the mean protein levels over the two measurements. Marginal effects, depicted as regression lines, were estimated for ages 30 to 60 by the 10^th^, 50^th^, and 90^th^ percentiles of the mean inflammatory protein levels.

Among the 79 replicated CpG sites, four displayed significantly altered DNA methylation levels within individuals over time (Figure 2). For one of these sites (cg08548559), methylation levels were associated with circulating levels of CRP, and three were associated with levels of CXCL9 and/or CXCL11 (cg09801824, cg16411857 and cg07839457). None of the within-individual changes over time were dependent on colorectal cancer case-control status (Supplementary Figure S1). An additional three CpG sites had temporal methylation trajectories with a suggestive dependency on protein levels (cg17501210 and cg10636246 associated with CRP, and cg05529343 associated with Flt3L, (Supplementary Figure S2). At cg17501210 (CRP-associated), methylation decreased over time in individuals with high CRP levels (90th percentile) at their first measuring occasion, whereas DNA methylation at the same site increased over time in individuals with low CPR levels (10th percentile) (P_interaction_ = 0.017). At cg10636246 (CRP-associated) and cg05529343 (Flt3L-associated), methylation decreased over time for individuals with protein levels in the 10^th^ percentile and increased for individuals in the 90^th^ percentile (P_interaction_ = 0.027 and 0.026, respectively, Supplementary Figure S2). Changes in these CpG sites were also independent of case-control status (Supplementary Figure S3).

### Relationship between DNA methylation and other inflammatory markers

**Figure 3.**
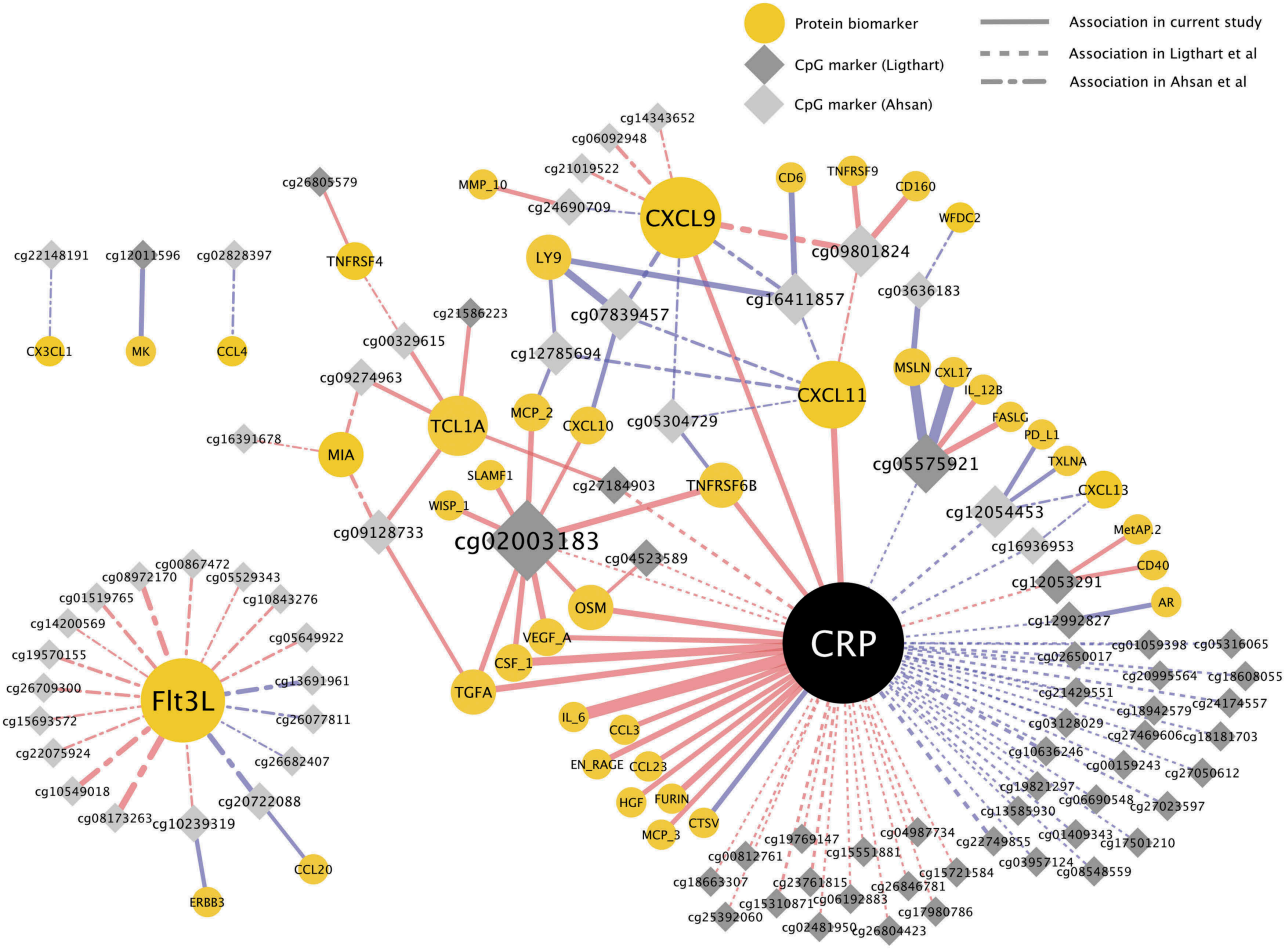
Network of associations between DNA methylation, CRP, and other protein biomarkers. Associations were estimated with mixed models, adjusted for age, sex, case-control status, BMI, smoking status, and technical covariates. Node size corresponds to number of associations (edges). Edge size corresponds to association strength measured as contribution to explained CpG methylation variance by adding the protein biomarker to the model. Edge color represents direction of association, red = positive, blue = negative association. Full network showing all Ligthart et al. [9] CRP-associated CpG sites and Ahsan et al. [10] protein-associated CpG sites available in the study, and all other associations with P < 0.0005 in the current study.

In order to illustrate the associations between CRP, 160 additional protein biomarkers measured in our dataset, and methylation at the 79 inflammation-associated CpG sites replicated in our study, we fitted mixed models and depicted the correlations as a network (Figure 3). CRP was, as expected, associated with multiple inflammatory markers, including interleukin 6 (IL-6), transforming growth factor alpha (TGFA), and several chemokines (CXCL3, CXCL11, and CXCL23) (Figure 3 and Supplementary Table 2). Associations between CpG sites and additional inflammatory markers (not included among the original 11 available for replication) were demonstrated for 19 sites. Ten of these were associated with at least three inflammatory markers, and 9 were associated with one. The CpG sites with associations to multiple inflammatory markers were situated at different loci throughout the genome, listed together with association results in Supplementary Table 3. Based on results from previous publications, none of the multi-associated markers had any genetic variants in proximity of the CpG marker with a methylation quantitative trait loci (mQTLs) effect [9,10].

Furthermore, we estimated associations between methylation levels at all CpG sites and plasma levels of all protein markers, and with metabolic and lifestyle variables (smoking, Swedish moist snuff use, BMI, alcohol intake, physical activity, blood pressure, fasting glucose levels, triglyceride levels and total cholesterol) (Supplementary Table 4). Methylation levels at three CPG sites were associated with smoking, all of which had been previously reported (cg05575921, cg03636183, cg16391678) [12,13]. Three CPG sites were associated with BMI (cg06192883, cg05304729 and cg14343652), of which one (cg06192883) was previously reported [14].

### Regional analyses of CpG sites associated with multiple proteins

**Figure 4.**
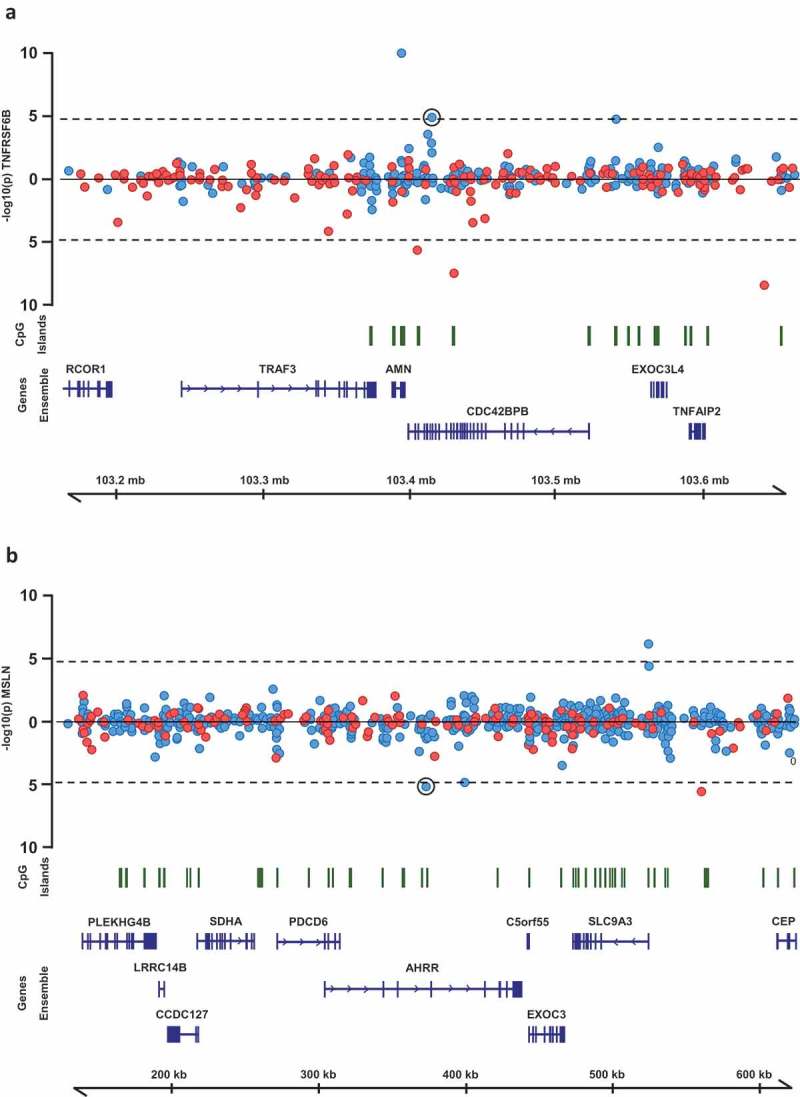
Regional association plots of CpG sites at which methylation levels were associated with multiple protein biomarkers. P-values from mixed models testing the association between (a) TNFRSF6B and CpG sites within 250kb of cg02003183 and (b) MSLN and CpG sites within 250kb of cg02003183. Points above the line represent positive associations between methylation and protein levels, and points below the line represent inverse associations. Blue points represent CpG sites on the Illumina 450k Infinium array, red points represent CpG sites on the Illumina 850k EPIC array.

For the ten CpG sites for which methylation levels associated with circulating levels of multiple inflammatory protein biomarkers (Supplementary Table 3), we performed regional analyses. All CpG sites within 250 kb of each original CpG were identified and their relationship with all proteins associated with the original CpG were assessed. Of 43 associations tested, two represented multiple adjacent CpG sites associated with one of the same proteins as the original CpG (Figure 4(a-b), Table 2). These included significant associations between methylation levels at five CpG sites near the original CpG cg02003183 at 14q32.32 (*CDC42BPB*) and circulating levels of TNFRSF6B. Interestingly, the most significant of these CpG sites (cg255277023) is situated in a predicted active promoter/enhancer region based on Combined Segmentations analysis from ENCODE (http://genome.ucsc.edu/encode/). The other region included four additional CpG sites surrounding the original CpG cg05575921 at 5p15.33 (*AHRR*), all of which were associated with circulating levels of mesothelin.

## Discussion

Alterations in DNA methylation resulting from inflammatory processes have previously been described [15] and represent a putative link between inflammation and downstream adverse health outcomes, including cancer [4]. This is further supported by epigenome-wide association studies in which methylation levels at multiple CpG sites were associated with circulating levels of CRP [9] or other inflammatory proteins [10]. However, many of these epigenetic changes are hypothesized to be under genetic control and may, therefore, not actually be due to inflammation [10]. Instead, genetic variants might control processes related to both inflammation and DNA methylation.

In this study, we used repeated samples from a population-based cohort to investigate previously published associations between DNA methylation, CRP and 11 additional inflammatory protein markers. In addition to confirming a majority of the associations, we also identified time-dependent changes that differed based on baseline levels of proteins. However, the potential impact of protein-associated DNA methylation sites may be small. The methylation changes are not large enough to warrant their use as inflammatory biomarkers, and they are unlikely to be solely responsible for gene expression changes resulting in fluctuating protein concentrations. Still, if replicated, these interactions might indicate that methylation levels at these sites are controlled by similar mechanisms as the inflammatory protein markers.

Using a network analysis to illustrate associations between CRP, other protein biomarkers and CpG sites we identified several central methylation sites, associated with multiple inflammatory proteins, possibly representing genetic enhancer regions. Two especially interesting regions are those surrounding CpG sites cg02003183 and cg05575921, which were associated with circulating levels of multiple proteins. The site cg02003183 is situated in the gene *CDC43BPB* coding for a serine/threonine protein kinase. Methylation levels at this site were associated with circulating levels of 10 different proteins including CRP, TGFA and TNFRSF6B. Interestingly, five additional CpG sites within 250Kb of cg02003183 were also associated with levels of TNFRSF6B, one of which (cg25577023, p = 1.1 × 10^−10^) was situated in predicted enhancer/promoter regions based on Combined Segmentations analysis from ENCODE. The ability of enhancers to regulate transcription of distant genes is well recognized [16], and our results support methylation as one way this regulation is applied.

TNFRS6B, also known as Decoy Receptor 3 (DcR3) is a glycosylated protein receptor lacking a transmembrane domain, which means that it only exists in soluble form [17]. It is part of the tumor necrosis factor receptor superfamily, which also includes tumor necrosis factor (TNF)-like cytokine 1A (TL1A) and the receptor DR3, all of which are highly upregulated under elevated inflammatory conditions and especially in inflamed intestinal tissue [18]. Corroborating its association with systemic inflammation, levels of TNFRS6B were also highly associated with levels of CRP.

The other interesting region identified in the network analysis surrounded cg05575921, situated in the gene *AHRR*, methylation of which is known for being associated with smoking status [19]. Aside from a previously reported association with CPR levels, we identified strong associations between DNA methylation levels at this site and levels of four additional proteins including mesothelin (coded for by the gene *MSLN*). Futhermore, surrounding cg05575921 were four additional CpG sites which also associated with meothelin levels. The MSLN gene is overexpressed in many cancers and likely to play a role in tumor progression [20].

It is also important to note that a majority of the novel associations in the network were connected to CRP levels. This can be explained by the overrepresentation of CRP in previous research, which provided the basis for our analyses. As such, the network cannot be seen as an unbiased association of interactions between proteins and DNA methylation, but an illustration of associations between robustly CRP-associated methylation sites and a large set of commonly studied inflammatory proteins.

The main strength of this investigation was the study design, with repeated, high-quality blood samples collected at standardized ages ten years apart. Furthermore, the samples were analyzed using the newest and largest methylation array available, together with large panels of protein biomarkers and extensive phenotype data, resulting in a unique data set useful for exploring novel associations. To aid comparisons between our study and previous results, as well as between all individual markers in our material, we modeled the methylation beta values as the outcome rather than exposure. However, theoretically it would also be plausible that changes to DNA methylation directly affect protein levels. Although the sample size was limited, the repeated samples and the strict inclusion criteria and sample handling improved the capacity to detect associations. We were able to account for several potential confounders, though the lack of data concerning aspirin and other anti-inflammatory drugs was a weakness of the study. Smoking and BMI could influence methylation through effects on inflammation, but probably also through other mechanisms. They were therefore included as potential confounders in our analyses, as in previous studies [9,10]. Other weaknesses include the large number of statistical comparisons in relation to the sample size, which made it more difficult to identify novel associations. However, our main exploratory analyses used previously reported CpG sites, reducing the need for replication. Another potential weakness is the original study population, which was collected as part of a study investigating pre-diagnostic biomarkers for colorectal cancer. However, all analyses were adjusted for colorectal cancer case status.

### Conclusions

Our study supports a complex interaction between DNA methylation and circulating proteins involved in the inflammatory response. The notion of trans-acting methylation sites affecting, or being affected by, the expression of genes on completely different chromosomes should be taken into account when interpreting results from epigenome-wide association studies.

## Materials and methods

### Study participants

This study is based on participants in the Västerbotten Intervention Programme (VIP), a large, ongoing, population-based cohort established in the late 1980’s, the details of which are provided elsewhere [21]. We included 138 individuals, all of whom had participated in the VIP on two occasions. The repeated samples were collected at ten year intervals for all participants except for two with a 20 year interval, and the large majority were collected at 50 and 60 years of age.

Participants were selected as part of a colorectal cancer study and included 69 cases and 69 control participants matched on sex, age (± 12 months) and sampling date (± 12 months). Cases were identified by linkage with the essentially complete Cancer Registry of Northern Sweden (ICD-10 18.0 and 18.2–18.9 for colon, 19.9 and 20.9 for rectum). The selection protocol required one sample from each case to have been collected within the five years preceding colorectal cancer diagnosis, excluding the final three months to avoid effects due to clinically manifest disease. Previous cancer other than non-melanoma skin cancer was also an exclusion criterion. The same restriction was applied to the controls, with the addition that they had to be cancer free at the latest follow-up in the study, 31 December 2014.

One participant was removed due to discordance between blood sampling and questionnaire date. Two participants were excluded due to identity mismatch between repeated samples in either the case or control (detected using the methylation data). Two participants had samples with probe call rate <96% at the detection P-value threshold 0.01 in the DNA methylation analysis, and were therefore also excluded. Eleven samples could not be analyzed in the targeted proteomic analysis due to technical problems. After all exclusions, the study included repeated samples from 127 participants (254 samples) with CRP and methylation data, and 118 participants (236 samples) with CRP, methylation, and proteomic data.

### Sample handling

All blood samples in this study were collected in in EDTA-tubes in the morning, after at least 8 hours of fasting. Samples were separated into buffy coat, plasma and erythrocyte fractions, aliquoted and frozen within one hour of collection, at −80°C directly or at −20°C for up to one week before transfer to a central storage facility. None of the samples had been thawed prior to aliquoting for the present study.

### DNA methylation analyses

Buffy coat DNA samples were bisulfite treated using the EZ DNA Gold Methylation kit from Zymo Research (Cat No: D5006) and analyzed for methylation using Infinium MethylationEPIC BeadChip (Illumina, Cat No; WG-317-1001). DNA quality control, pre-processing, processing and output data quality control were performed at the SNP&SEQ Technology Platform, Uppsala, Sweden, part of the National Genomics Infrastructure (NGI) Sweden and Science for Life Laboratory. DNA methylation data was preprocessed using the ENmix and minfi R-package [22,23]. Before preprocessing, SNP-related probes (SNP list from [24]), probes with call rate P-value<0.01 and probes with missing in more than 20% of samples were removed (3094 probes). Background correction of methylation signal intensities was made with the ENmix-algorithm. We applied inter-array normalization with the quantile method and probe-type bias adjustment using the BMIQ-method. Methylation at each CpG site was represented as a β value, calculated as the proportion of methylated signal intensity out of total signal intensity, ranging 0 to 1 indicating zero to 100% methylation. White blood cell proportions were estimated based on the β values using the Houseman method [25], using the *estimateCellCounts* function in the minfi package. As the Hausman method was developed for the 450k methylation array, estimates based on the EPIC array utilize probes common to both the EPIC and 450k methylation array. Comparisons have shown that estimated proportions are very similar to using all 450k probes [22].

### Protein analysis

Concentrations of CRP were measured in pre-coated 96-well plates using the V-PLEX Human CRP Kit (Meso Scale Discovery, cat no: K151STD) and according to the manufacturer’s instructions summarized here in brief. All samples were diluted 1/1000, after which 25μl sample dilution was added to the pre-coated plates. Standards and pooled plasma controls were added in duplicate to all plates. Plates were incubated on a shaker at room temperature for two hours. Detection antibody solution was added to the plates, which were incubated for an additional hour on a shaker. Reading Buffer was then added and plates were immediately read on a MESO QuickPlex SQ 120 (MSD, Cat No: AI0AA-0). Plates were washed 3 times between all steps. Matched case sets were analyzed together, in random order, on the same analysis plate. Investigators and laboratory staff were blinded to case and control status up until data preprocessing and analyses.

Inter- and intra-assay coefficients of variation (CVs), calculated on control samples, were 1 and 0.4% respectively.

Targeted proteomic analyses of 178 plasma proteins (Olink Oncology II and Olink Inflammation panels) were conducted by multiplex immunoassay, using proximity extension assay technology at Olink Proteomics, Uppsala, Sweden (Supplementary Table 5). Processing, output data quality control, and normalization were performed by Olink Proteomics. Data were delivered as Normalized Protein eXpression (NPX) values on a log2 scale. Data values below the LOD were assumed to be missing, and proteins with >50% missing values were excluded (IL-20RA, IL-2RB, IL-1-alpha, IL-2, TSLP, IL-10RA, IL-22.RA1, IL-24, IL-13, ARTN, TNF, IL-20, IL-33, IFN-gamma, IL-4, LIF, NRTN, and IL-5), leaving 160 proteins for further analysis.

### Variables

In addition to blood samples, extensive health and lifestyle data are collected in the VIP. The variables used in this study were case status (colorectal cancer case/control), sex (male/female), age at sampling (years, continuous), body mass index based on weight and height measurements taken by a health professional (BMI, kg/m^2^), mid-blood pressure (mean of systolic and diastolic blood pressure, mmHg), and fasting blood glucose (mmol/l), total cholesterol (mmol/l), and triglyceride levels (mmol/l). Furthermore, we included questionnaire-based lifestyle data on recreational physical activity (1–5 scale from never to more than three times/week), tobacco smoking (never-, current, and ex-smoker), Swedish moist snuff tobacco (snus) use (never-, current, and ex-user), and intake of alcohol (grams/day) estimated from food frequency questionnaires.

### Statistical analyses

Associations between CpG markers and plasma CRP or other proteins were estimated in linear mixed models using the lme4 R-package [26]. All models included log-transformed protein levels, age, sex, case-control status, BMI, smoking status, white blood cell type proportions, the first principle component of the β-values as fixed factors, and participant identification number as a random factor. Variance explained by fixed factors ($R_{m}^{2}$) were estimated using the MuMin R-package [27]. We evaluated the consistency of direction of effect in CpG -protein associations between our study and previous studies by calculating the proportion of regression coefficients with the same direction, and testing with a binomial test the null hypothesis that the true proportion was equal to 0.5 (i.e., no correlation between association in our study and previous study). To evaluate whether within-individual age trajectories for methylation differed by levels of CRP or other inflammatory proteins, we estimated linear mixed models including interaction terms between age and mean intra-individual protein levels. Associations were tested using t-tests of regression coefficients equal to zero using Satterthwaite’s approximation of degrees of freedom. Missing values for the lifestyle variables were set to the value from the other sampling occasion. Missing values for the protein biomarkers were excluded separately in each analysis (complete case analysis).

Cross-biomarker associations between the CpG sites, plasma CRP, and other inflammatory or cancer-related proteins were estimated using linear mixed models as described above. All CpG sites (replicated and non-replicated Ligthart et al. [9] and Ahsan et al. CpG sites [10]) were fitted against all protein biomarkers (CRP and 160 proteins, including the 11 in Ahsan et al.), and CRP was in turn fitted against all protein biomarkers. Associations between CpG sites and CRP with the same direction as in Ligthart et al. [9], as well as the top associations among all inter-biomarker associations according to P-value (P < 0.0005), were plotted in a network.

For CpG sites at which methylation levels were associated with three or more protein markers, we performed regional analyses, estimating associations to the same protein for all CpG markers within ±250 kb of the CPG site. This approach was applied, rather than an agnostic method, due to the limited sample size. The results were visualized using the Gviz R-package [28]. Genomic annotation features in hg19 were extracted from the Ensembl data base. Only gene models included in NCBI Reference Sequence Database were included. Transcripts were collapsed into single genes containing the exons of all transcripts for visualization purposes.

All tests were 2-sided when applicable. For analyses not strictly confirmatory, we used a significance threshold of p < 0.0005. For the regional analyses, we used a Bonferroni-adjusted significance threshold of p < 0.05 based on the number of analyzed CpG sites (0.05/3082 ≈ 1.6*10^−5^).
