# Supplementary material for: Circulating levels of inflammatory markers and DNA methylation, an analysis of repeated samples from a population based cohort
Source: Epigenetics. 2019 Apr 29;14(7):649–59. doi: 10.1080/15592294.2019.1603962 (PMC6557598; doi:10.1080/15592294.2019.1603962)
Supplement: Supplemental Material [file kepi-14-07-1603962-s001.docx]

Supplementary Figure S1


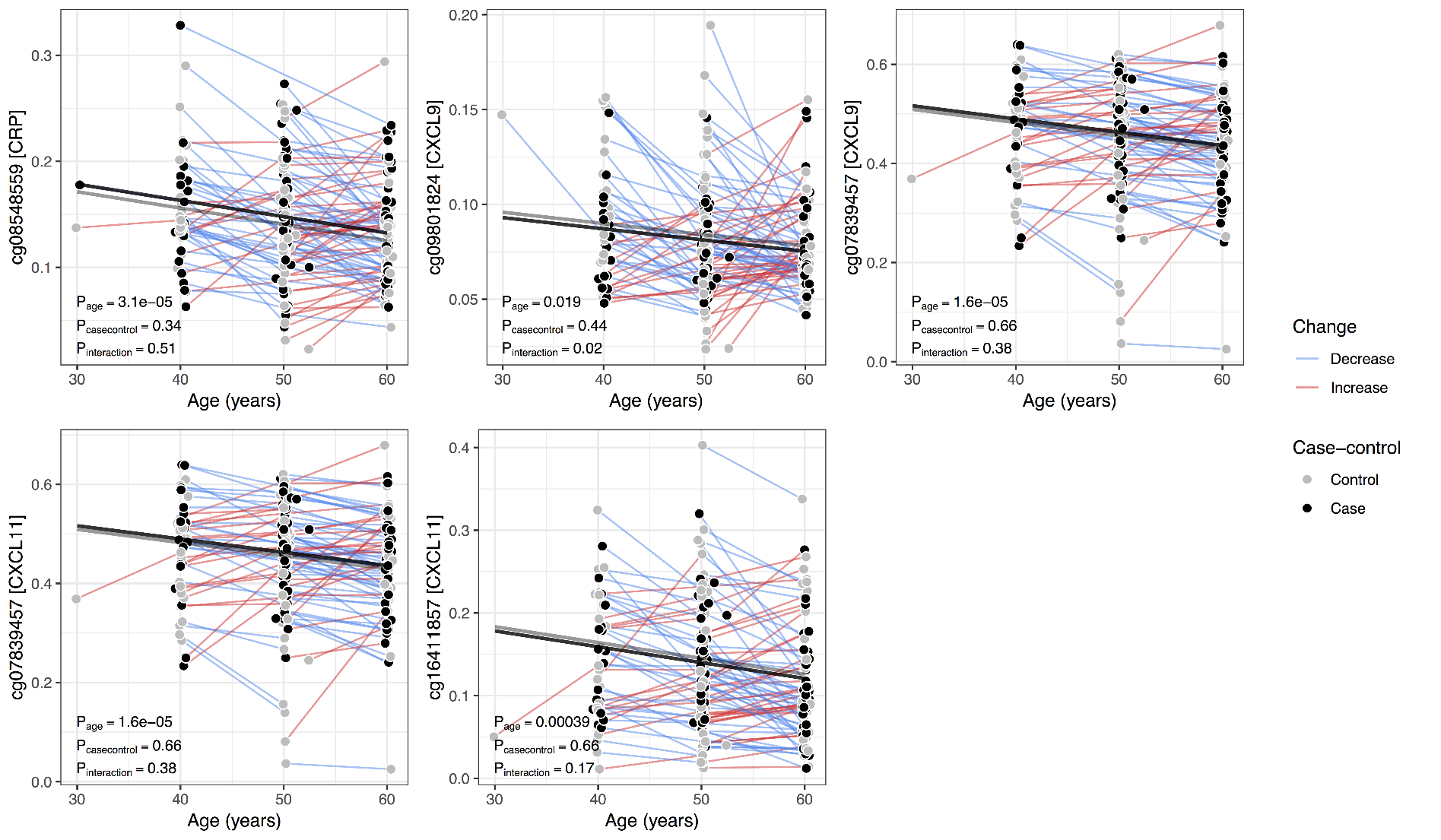


**Figure S1. Average within-individual age trajectories for CpG methylation levels by colorectal cancer case-control status.** The CpG sites shown demonstrated an association between methylation and levels of an inflammatory protein (in square brackets on the y-axis), as well as a significant change over time (P<0.0005). Average within-individual age trajectories were estimated in mixed models, including interaction terms between age and case-control status. Marginal effects, depicted as regression lines, were estimated for ages 30 to 60 by case-control status.

Supplementary Figure S2


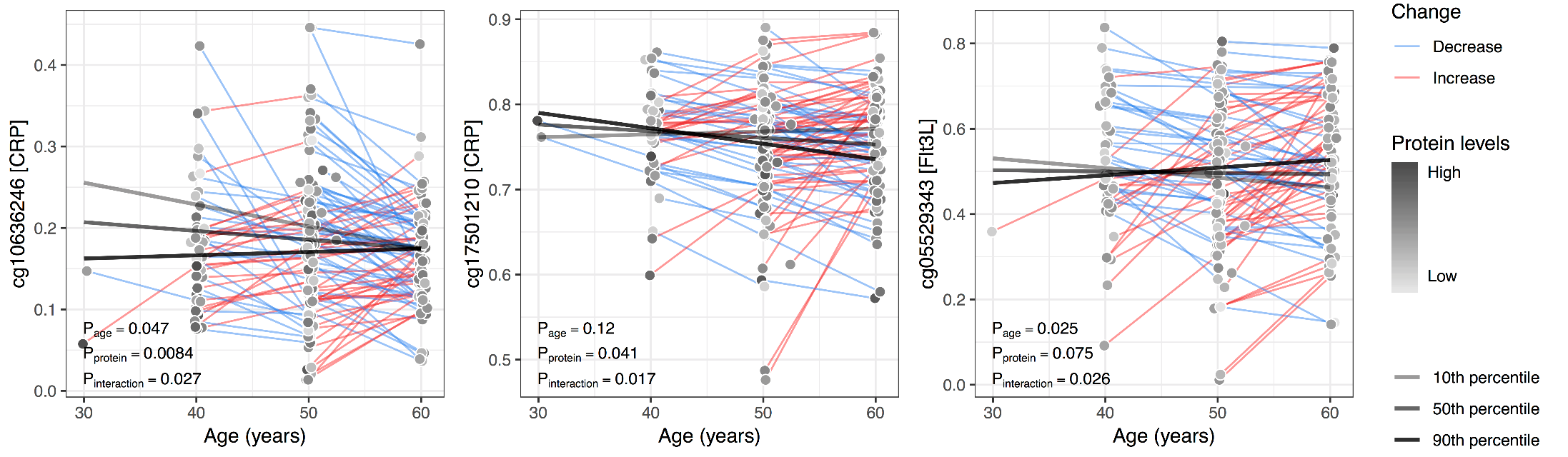


**Figure S1. Average within-individual age trajectories for CpG sites with suggested dependency on protein levels.** The CpG sites shown demonstrated potential interactions between methylation levels and levels of an inflammatory protein (in square brackets on the y-axis). Average within-individual age trajectories were estimated in mixed models, including interaction terms between age and mean protein levels over the two measurements. Marginal effects, depicted as regression lines, were estimated for ages 30 to 60 by the 10^th^, 50^th^, and 90^th^ percentiles of the mean inflammatory protein levels.

Supplementary Figure S3


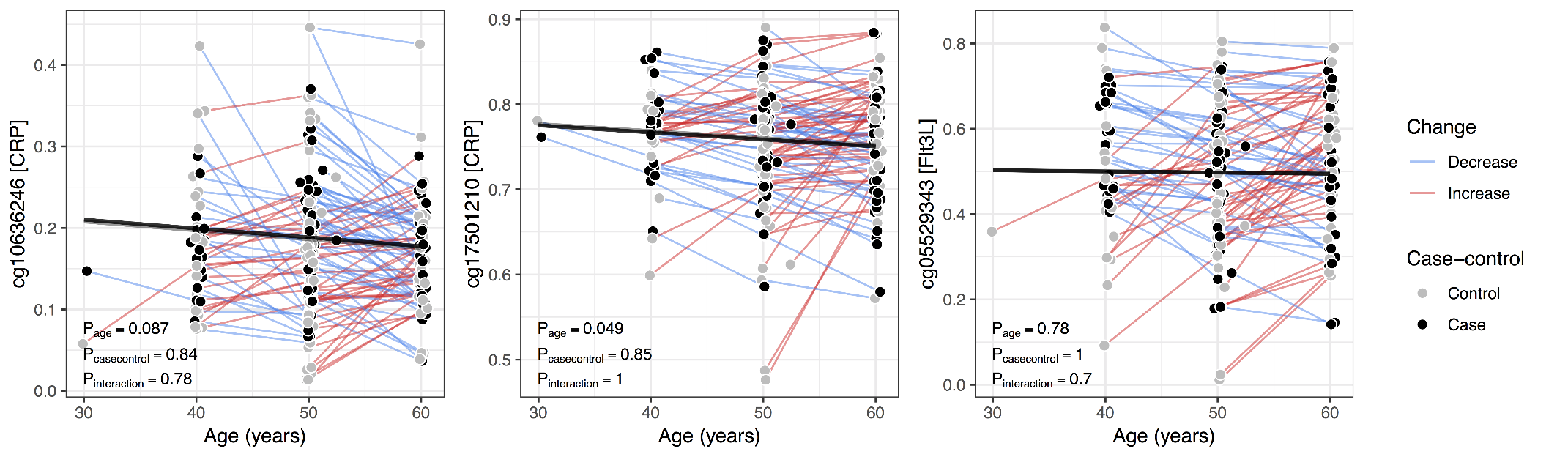


**Figure S3. Average within-individual age trajectories for CpG sites with suggested dependency on protein levels by colorectal case-control status.** The CpG sites shown demonstrated potential interactions between methylation levels and levels of an inflammatory protein (in square brackets on the y-axis). Average within-individual age trajectories were estimated in mixed models, including interaction terms between age and case-control status. Marginal effects, depicted as regression lines, were estimated for ages 30 to 60 by case-control status.
